# Mapping the binding sites of antibodies utilized in programmed cell death ligand-1 predictive immunohistochemical assays for use with immuno-oncology therapies

**DOI:** 10.1038/s41379-019-0372-z

**Published:** 2019-09-26

**Authors:** Nicola L. Lawson, Carly I. Dix, Paul W. Scorer, Christopher J. Stubbs, Edmond Wong, Liam Hutchinson, Eileen J. McCall, Marianne Schimpl, Emma DeVries, Jill Walker, Gareth H. Williams, James Hunt, Craig Barker

**Affiliations:** 1 Precision Medicine, Oncology R&D, AstraZeneca, Cambridge, UK; 2 Antibody Discovery and Protein Engineering (ADPE), R&D, AstraZeneca, Cambridge, UK; 3 Structure, Biophysics and Fragment-Based Lead Generation, Discovery Sciences, BioPharmaceuticals R&D, AstraZeneca, Cambridge, UK; 4 Spirogen, AstraZeneca, London, UK; 5 FM Operations, BioPharmaceuticals R&D, AstraZeneca, Cambridge, UK; 6 Precision Medicine, Oncology R&D, AstraZeneca, Cambridge, UK; 7 Oncologica UK Ltd, Cambridge, UK

**Keywords:** Predictive markers, Cancer immunotherapy

## Abstract

Programmed cell death ligand-1 (PD-L1) expression levels in patient tumor samples have proven clinical utility across various cancer types. Several independently developed PD-L1 immunohistochemical (IHC) predictive assays are commercially available. Published studies using the VENTANA PD-L1 (SP263) Assay, VENTANA PD-L1 (SP142) Assay, Dako PD-L1 IHC 22C3 pharmDx assay, Dako PD-L1 IHC 28-8 pharmDx assay, and laboratory-developed tests utilizing the E1L3N antibody (Cell Signaling Technology), have demonstrated differing levels of PD-L1 staining between assays, resulting in conjecture as to whether antibody-binding epitopes could be responsible for discordance between assays. Therefore, to understand the performance of different PD-L1 predictive immunohistochemistry assays, we aimed to distinguish the epitopes within the PD-L1 protein responsible for antibody binding. The sites at which antibody clones SP263, SP142, 22C3, 28-8, and E1L3N bind to recombinant PD-L1 were assessed using several methods, including conformational peptide array, surface plasmon resonance, and/or hydrogen/deuterium exchange mass spectrometry. Putative binding sites were confirmed by site-directed mutagenesis of PD-L1, followed by western blotting and immunohistochemical analysis of cell lines expressing mutant constructs. Our results demonstrate that clones SP263 and SP142 bind to an identical epitope in the cytoplasmic domain at the extreme C-terminus of PD-L1, distinct from 22C3 and 28-8. Using mutated PD-L1 constructs, an additional clone, E1L3N, was also found to bind to the cytoplasmic domain of PD-L1. The E1L3N binding epitope overlaps considerably with the SP263/SP142 binding site but is not identical. Clones 22C3 and 28-8 have binding profiles in the extracellular domain of PD-L1, which differ from one another. Despite identifying epitope binding variance among antibodies, evidence indicates that only the SP142 assay generates significantly discordant immunohistochemical staining, which can be resolved by altering the assay protocol. Therefore, inter-assay discordances are more likely attributable to tumor heterogeneity, assay, or platform variables rather than antibody epitope.

## Introduction

Immunotherapies targeting programmed cell death-1 (PD-1) or programmed cell death ligand-1 (PD-L1) pathways offer a novel treatment avenue for patients with cancer [1]. Five PD-1/PD-L1 immunotherapies (atezolizumab, avelumab, durvalumab, nivolumab, and pembrolizumab) are approved by the Food and Drug Administration and/or European Medicines Agency for multiple indications [1, 2]. The interaction between PD-1, a cell-surface receptor expressed on T cells and activated B cells, and its ligand PD-L1, a member of the B7 family of cell-surface ligands that regulates T-cell activation and immune response, plays a crucial role in inhibiting the body’s immune response to foreign antigens [2, 3]. Many cancer cell types express PD-L1 and thereby activate PD-1/PD-L1 signaling, thus enabling the tumors to evade immune recognition [3]. Responsiveness to anti-PD-1 antibodies such as pembrolizumab and nivolumab or to anti-PD-L1 antibodies atezolizumab, durvalumab, and avelumab may be predicted by the PD-L1 expression on tumor cells (TCs) and/or tumor-infiltrating immune cells (ICs) [3, 4]. Thus, PD-L1 expression level is a predictive biomarker for the efficacy of anti-PD-1/PD-L1 therapies and guides patient selection for these therapies [3].

Multiple PD-L1 immunohistochemistry (IHC) tests are marketed as companion or complementary assays for use with individual anti-PD-L1 therapies (Table 1) [5–12]. The VENTANA PD-L1 (SP263) Assay is approved for use with durvalumab treatment in urothelial carcinoma (UC) and non-small-cell lung cancer (NSCLC) and with pembrolizumab and nivolumab treatment in non-small-cell lung carcinoma [5, 6]. The VENTANA PD-L1 (SP142) Assay is approved for use with atezolizumab treatment in non-small-cell lung carcinoma and UC [7, 8]. The Dako PD-L1 IHC 28-8 pharmDx and Dako PD-L1 IHC 22C3 pharmDx are approved for use with nivolumab and pembrolizumab treatment, respectively, in multiple cancer types [9–12]. These assays differ in the antibody clones, immunohistochemistry protocols, scoring algorithms, and tumor cell and immune cell cutoffs for PD-L1 positivity [13]. The information available on the epitopes bound by PD-L1 antibodies utilized in predictive immunohistochemistry assays is limited [14, 15].

**Table 1:** Comparison of PD-L1 assays

PD-L1 assay	Approval status	Indication	IO therapy and cutoff
VENTANA PD-L1 (SP263) Assay [5, 6]	FDA and CE-IVD approved for UC and CE-IVD approved for NSCLC	NSCLC UC	Durvalumab: TC ≥ 1% post chemoradiation therapy Pembrolizumab: TC ≥ 50% in first line; TC ≥ 1% in second line Nivolumab: TC ≥ 1%,≥5%, and≥10% in second line Durvalumab: TC ≥ 25%; or ICP > 1% and IC ≥ 25%; or ICP = 1% and IC = 100% in second line
VENTANA PD-L1 (SP142) Assay [7, 8]	FDA and CE-IVD approved	NSCLC UC	Atezolizumab: TC ≥ 50% or IC ≥ 10% Atezolizumab: IC ≥ 5%
Dako PD-L1 IHC 28-8 pharmDx [9, 10]	FDA and CE-IVD approved	NSCLC UC HNSCC Melanoma	Nivolumab: TPS ≥ 1%, ≥5%, ≥10% Nivolumab: TPS ≥ 1% Nivolumab: TPS ≥ 1% Nivolumab: TPS ≥ 1%
Dako PD-L1 IHC 22C3 pharmDx [11, 12]	FDA approved for NSCLC, gastric adenocarcinoma, UC, and cervical cancer CE-IVD approved for melanoma, NSCLC, and UC	NSCLC UC Gastric adenocarcinoma Cervical carcinoma	Pembrolizumab: TPS ≥ 1% or TPS ≥ 50% Pembrolizumab: CPS ≥ 10 Pembrolizumab: CPS ≥ 1 Pembrolizumab: CPS ≥ 1

*CE* Conformité Européenne, *CPS* combined positive score, *FDA* Food and Drug Administration, *HNSCC* head and neck squamous cell carcinoma, *IC* immune cell, *ICP* immune cells present, *IVD* in vitro diagnostic device, *IHC* Immunohistochemistry, *UC* urothelial carcinoma, *NSCLC* non-small cell lung cancer, *TC* tumor cell, *TPS* tumor proportion score

Structurally, PD-L1 is a 290-amino acid transmembrane glycoprotein composed of two extracellular immunoglobulin (Ig) domains (V1-type and C2-type) and a 31-residue cytoplasmic domain [16, 17]. PD-L1 is found to be N-glycosylated at N_35_, N_192_, N_200_, and N_219_ residues [18]. Both SP263 and SP142 antibody clones reportedly bind amino acid residues 284–290 in the cytoplasmic tail of PD-L1 [6, 8, 19–21]. However contrary to these reports, several recent publications have referred to the epitope for the SP263 antibody as being located in the extracellular PD-L1 domain [22–25]. The 28-8 antibody reportedly targets the extracellular domain of mature human PD-L1 (residues 19–239), but no specific data on its binding epitope are available [10, 26]. The putative epitope for the 22C3 antibody is reported to be two discontinuous segments in the extracellular PD-L1 domain by hydrogen/deuterium exchange mass spectrometry (HDX-MS) epitope mapping; residues within regions 156–178 and 196–206 in PD-L1 showed strong protection from deuteration [27]. In addition, residues within regions 3–9, 10–13, 88–93, and 135–147 in PD-L1 showed marginal yet significant protection and were also identified as contributing to the epitope for the 22C3 antibody [27]. Varying levels of concordance reported between PD-L1 assays [28, 29] have resulted in conjecture as to whether the antibody-binding epitope is one of the factors contributing to the discordance [23, 30–32]. A study involving the standardization of PD-L1 immunohistochemistry antibodies demonstrated that the antibodies were indeed highly concordant, suggesting that variations in PD-L1 staining were independent of the antibody used and likely attributable to assay platform functionality, tumor heterogeneity, or other factors [14]. Efforts continue to be made to compare staining and scoring algorithms across these immunohistochemistry assays in an attempt to gain insight into the potential for assay harmonization in clinical testing [33, 34]. It is important to investigate whether the epitopes of antibodies involved in PD-L1 immunohistochemistry assays also contribute to the assay discordance.

This study aims to characterize in detail the epitopes within the PD-L1 protein bound by each of the commonly used PD-L1 antibodies utilized in approved immunohistochemistry assays, using multiple techniques, and provide an informed assessment of the degree to which antibody epitope binding influences PD-L1 immunohistochemistry assay concordance.

## Materials and methods

### Linear and discontinuous epitope mapping by peptide array

An extensive library of linear peptides for continuous epitopes and nonlinear, discontinuous, conformationally constrained, peptide mimics corresponding to the PD-L1 extracellular domain (residues 19–239) and the cytoplasmic domain (residues 260–290) was designed and used to create a peptide array by the Chemical Linkage of Peptides on Scaffolds (CLIPS; Pepscan Presto, Netherlands) technique [35, 36]. All peptides were synthesized on solid support and screened with varying concentrations of antibody and blocking buffer to optimize signal-to-noise ratios in each experiment. Arrays were probed with SP263 (790-4905; Ventana Medical Systems, Inc., USA), SP142 (M4422; Spring BioScience, USA), 22C3 RTU (SK006; Dako, Denmark), 28-8 (ab205921; Abcam, UK), and recombinant 22C3 and 28-8 antibodies described in the following section. The binding of antibodies to each peptide construct of the entire library was determined.

### Recombinant 22C3 and 28-8 antibody production, purification, and antigen-binding fragment (Fab) generation

Recombinant versions of antibodies 28-8 (r28-8) and 22C3 (r22C3) were created by cloning the reported variable heavy-chain and variable light-chain sequences [26, 27] into rabbit and mouse IgG expression vectors, respectively. The full sequences of the IgGs produced are presented in Supplementary Table S1. A high-yielding Chinese Hamster Ovary (CHO) transient expression system with suspension-adapted CHO-K1 cells engineered to express the Epstein–Barr virus nuclear antigen-1 with co-expression of the glutamine synthetase gene (CHO-EBNA-GS) cells was obtained from MedImmune (Cambridge, UK) [37]. r28-8 and r22C3 monoclonal antibodies were expressed and purified from CHO-EBNA-GS cells for probing peptide arrays, surface plasmon resonance (SPR), hydrogen/deuterium exchange mass spectrometry, western blot, and confocal microscopy experiments. Detailed protocols for generating full-length and antigen-binding fragments of r28-8 and r22C3 antibodies are presented in Supplementary Appendix A.

### Surface plasmon resonance

Binding kinetics of 28-8 and 22C3 antibodies to PD-L1 protein were analyzed by SPR using r28-8, r22C3, and a His-tagged extracellular domain of PD-L1 (residues 19–238; provided by MedImmune, Cambridge, UK). SPR experiments were performed using a Biacore T200 instrument (GE Healthcare, Sweden) at 25 °C in a running buffer composed of phosphate-buffered saline (PBS; 10 mM sodium phosphate, 154 mM NaCl, pH 7.4; Sigma Aldrich, USA) supplemented with 0.05% (v/v) Tween-20. All samples were diluted in running buffer. A Series S Sensor Chip C1 was functionalized using a mouse antibody capture kit (BR100838; GE Healthcare, Sweden) as per the manufacturer’s instructions, resulting in 950 resonance units (RU) of anti-mouse antibody captured on the surface. For r22C3 binding experiments, 90 RU of r22C3 antibody was captured on the surface by injecting 1 µg/mL r22C3 for 120 s at 1 μL/min in each analyte cycle; PD-L1 was injected at different concentrations (top concentration 110 nM, two-fold serial dilution) for 240 s with a dissociation time of 1200 s. For r28-8 binding experiments, 90 RU of r22C3 antibody was captured on the surface, then PD-L1 was first injected for 240 s at 110 nM, followed by increasing concentrations of r28-8 antibody (top concentration 33 nM, two-fold serial dilution) injected for 120 s with a dissociation time of 300 s. After each analyte cycle, the surface was regenerated with 3 × 120 s injections of 10 mM glycine-HCl (pH 1.7). Kinetic rate constants were derived by fitting the data to a Langmuir 1:1 ligand binding model provided by the Biacore T200 evaluation software (version 2, GE Healthcare, Sweden).

### Hydrogen/deuterium exchange mass spectrometry

The extracellular domain of mature PD-L1 was incubated either alone or in complex with antigen-binding fragments of r28-8 and r22C3 and subjected to hydrogen/deuterium exchange mass spectrometry. Deuterium exchange reactions were performed using a LEAP H/D-X PAL system (LEAP Technologies, USA) and all experiments were performed in duplicate. PD-L1 (10 μM) was incubated with or without Fab (20 μM) in 2 × Dulbecco’s PBS (21600010; Gibco, USA) at 4 °C. Deuterium exchange reactions were initiated by diluting the protein solutions 1:10 to a final volume of 50 μL in 1 × Dulbecco’s PBS prepared in D_2_O (99.9% atom D, Sigma Aldrich, USA), and incubating at 20 °C for 30, 300, 900, 3600, and 7200 s. Undeuterated controls were prepared by performing the same dilution in 1 × Dulbecco’s PBS prepared in water. Labeling reactions were quenched by transferring 50 μL of reaction mixture to 50 μL of pre-chilled quench solution (3.0 M urea, 1.6% [v/v] formic acid in water; 4 °C). Quenched samples were directly injected onto an Enzymate BEH immobilized pepsin column (3 μm, 2.1 mm × 300 mm; Waters, USA) at 200 μL/min at 20 °C for 2 min. Peptic peptides were trapped and desalted on an ACQUITY UPLC BEH C18 VanGuard Pre-column (130 Å, 1.7 μm, 2.1 mm × 5 mm; Waters, USA) kept at 0.1 °C. The trapped peptides were eluted over 11 min using a 10–40% gradient of acetonitrile in 0.1% (v/v) formic acid at 38 μL/min on an Acquity UPLC BEH C18 column (130 Å, 1.7 μm, 50 mm × 1 mm; Waters, USA) at 0.1 °C. Peptides were detected on a SYNAPT G2-Si HDMS mass spectrometer (Waters, USA) acquiring over a mass-to-charge ratio (m/z) range of 50–2000 with an electrospray source and lock mass calibration (leucine enkephalin, 50 pg/μL; Waters, USA). The mass spectrometer was operated at a source temperature of 60 °C and a spray voltage of 3.0 kV. Spectra were collected in positive ion mode.

Peptide identification was performed in Protein Lynx Global Server (Waters, USA) using MS^E^ data collected for the undeuterated control samples. The resultant peptide lists were imported into DynamX (Waters, USA) and were filtered as follows: minimum intensity of 5000, minimum of 0.2 products per amino acid, a maximum MH+ error of 5 ppm, and found in >50% of the data sets. The automatic peptide assignment in DynamX was performed using the standard parameters, but all peptides were manually checked for the charge state assignment, overlapping peptides, and retention time. Data were not corrected for back-exchange; hence, deuterium uptake values were relative and not absolute. The relative deuterium uptake values generated by DynamX were considered statistically significant if greater than 0.5 Da. Significant differences were subsequently averaged over different time points, and data from redundant peptides were used to increase the resolution of the hydrogen/deuterium exchange mass spectrometry measurements. The hydrogen/deuterium exchange mass spectrometry data were mapped onto the X-ray structures of PD-L1 using PyMOL with coordinates taken from Protein Data Bank accession code 3FN3 [38].

### Immunofluorescence microscopy

Detailed protocol for immunofluorescence microscopy can be found in Supplementary Appendix B.

### PD-L1 mutants

Based on peptide array and hydrogen/deuterium exchange mass spectrometry data, single or multiple amino acid mutations with minimal likelihood of protein structure disruption were designed within the putative epitopes for SP263/SP142, 22C3, and 28-8. Expression vectors containing the complementary DNA of mutant PD-L1 constructs (pcDNA-DEST47 and pcDNA3.1) were purchased from GeneArt (Life Technologies, USA) and generated by site-directed mutagenesis in the putative binding sites of PD-L1 for SP263/SP142 (Int1–Int7) and for 22C3 and 28-8 (Ext1–Ext13), in addition to PD-L1 and programmed cell death ligand-2 (PD-L2) wild-type constructs (Supplementary Table S2). A construct containing the extracellular domain of PD-L2 fused to the last 27 residues of the PD-L1 cytoplasmic domain (P2-P1) was also generated. PD-L1 mutants were expressed in human Expi293F cell line (A14527; Life Technologies, USA), adapted to serum-free culture media and growth in suspension, and cultured in shake flasks in Expi293 expression media (A14351; Life Technologies, USA) at 250 rpm and 37 °C with 8% CO_2_. Detailed protocols for expression and western blotting of PD-L1 mutants are available in Supplementary Appendix C and D.

### Immunohistochemistry

Expi293F cells transfected with PD-L1 and PD-L2 constructs, as described previously, were harvested 48 h post-transfection. Cells were centrifuged and washed twice in PBS, followed by resuspension and fixation in 10% neutral buffered formalin for 30 min (9713.1000; VWR, USA). Following fixation, cells were centrifuged and resuspended in agarose (R0801; Thermo Scientific, USA). Solidified agarose cell cores were processed overnight in an Excelsior AS Tissue Processor (Thermo Scientific, USA). Cell cores were fixed in 10% formalin for 30 min, dehydrated in ethanol (6 × 1 h) and xylene (6 × 1 h), followed by paraffin wax (3 × 1 h 20 min), and finally embedded in paraffin blocks.

Formalin-fixed, paraffin-embedded (FFPE) cell lines and tissue blocks were sectioned at 4 µm and mounted on positively charged slides (S21.2113.A; Leica Bond Plus, Germany). Sections were dried and subsequently adhered to slides by baking at 60 °C for 1 h. Immunohistochemical staining was performed on automated platforms as per manufacturer’s instructions (SP263 and SP142 assays on Ventana Benchmark Ultra and Dako 22C3 pharmDx and Dako 28-8 PharmDx assays on Dako Link 48) at Hematogenix (Tinley park, IL, USA). Staining with E1L3N and PD-L2 was performed on a Leica Bond RX automated staining instrument using the Bond Polymer Refine Detection System IHC protocol F (DS9800; Leica Biosystems, UK) with the following modifications: E1L3N—20-min heat-induced epitope retrieval using ER2, 20-min protein block, and 30-min primary antibody incubation at 1/500 dilution; PD-L2—30-min heat-induced epitope retrieval using ER2 and 30-min primary antibody incubation at 1/25 dilution. Following staining, slides were dehydrated through graded alcohols, coverslipped, and mounted with ClearVue XYL mounting medium (4212; Thermo Scientific, USA). Tonsil and placenta tissues were used as positive controls and untransfected FFPE Expi293F cells served as a negative control. Stained slides were digitally scanned using the Aperio ScanScope AT2 slide scanner (Leica Biosystems, Germany), visualized using ImageScope software, and scored microscopically by a single qualified pathologist (Prof. Gareth Williams, Oncologica UK Ltd, Cambridge, UK) using a semiquantitative *H*-score system.

### Deglycosylation and mass spectrometry of deglycosylated PD-L1

Purified PD-L1 extracellular domain was deglycosylated by treatment with PNGase F (New England Biolabs, USA) for 1 h, as described by the manufacturer. Removal of N-linked oligosaccharide was confirmed by sodium dodecyl sulfate polyacrylamide gel electrophoresis (SDS-PAGE). To determine whether antibody binding was affected by deglycosylation, 500 ng of glycosylated or deglycosylated sample was reduced, denatured, and analyzed by western blotting, as described in Supplementary Appendix D, with 22C3 and 28-8 antibodies.

Detailed protocols for sample preparation and mass spectrometry analysis of deglycosylated PD-L1 are described in Supplementary Appendix E.

## Results

### r22C3 and r28-8 antibodies

r22C3 and r28-8 antibodies were verified by SDS-PAGE by comparing the reduced and nonreduced antibody samples. Due to the poor visibility of the light chain for r28-8, the antibody was compared with the commercially obtained 28-8 antibody (Supplementary Fig. 1A and 1B). The commercial and recombinant 28-8 antibodies demonstrated similar profiles on SDS-PAGE. To further validate the identity of r22C3 and r28-8, nonreduced bands corresponding to the antibodies were excised from the SDS-PAGE gel. Samples were reduced, alkylated, and digested with trypsin. The resultant digests were subjected to mass spectrometry. The data was processed using BioPharmaView software and verified manually against the unique peptide sequences of the heavy and light chain complementarity determining regions of 22C3 and 28-8.

### SP263 and SP142 mapped to epitopes in the cytoplasmic domain of PD-L1 using peptide array

Linear and discontinuous epitope mapping using peptide arrays demonstrated that both SP263 and SP142 mapped to an identical linear sequence (_284_DTHLEET_290_) in the cytoplasmic domain of the full-length PD-L1 protein (Supplementary Fig. S2). As signals obtained with the prediluted antibody provided with 22C3 PharmDx assay were too low for reliable epitope mapping, r22C3 was substituted to allow increased concentrations to be used for epitope mapping. Under moderately stringent conditions, r22C3 did not bind to any peptide, whereas 28-8 (at 0.5 µg/mL) mapped to peptides containing the stretch _205_EIFYCTFRR_213_, which lies in the extracellular C2 domain of PD-L1. With increased antibody concentration, 28-8 (at 2.5 µg/mL) bound to additional peptide stretches _205_EIFYCTFRRLD_215_ and _154_TCQAEGYPKAEVIWT_168_ (Supplementary Fig. S2). These two peptides are linked by a disulfide bridge in the C2 domain, suggesting that the epitope for 28-8 binding is discontinuous; with binding predominantly reliant on residues in the region 205–215 and complemented by contributions from residues in the region 154–168. As discontinuous 28-8 mapping resulted in low signals compared with linear epitope mapping, additional methods were used to confirm the discontinuous epitopes.

### r22C3 and r28-8 antibodies bind independently to the extracellular domain of PD-L1

**Fig. 1:**
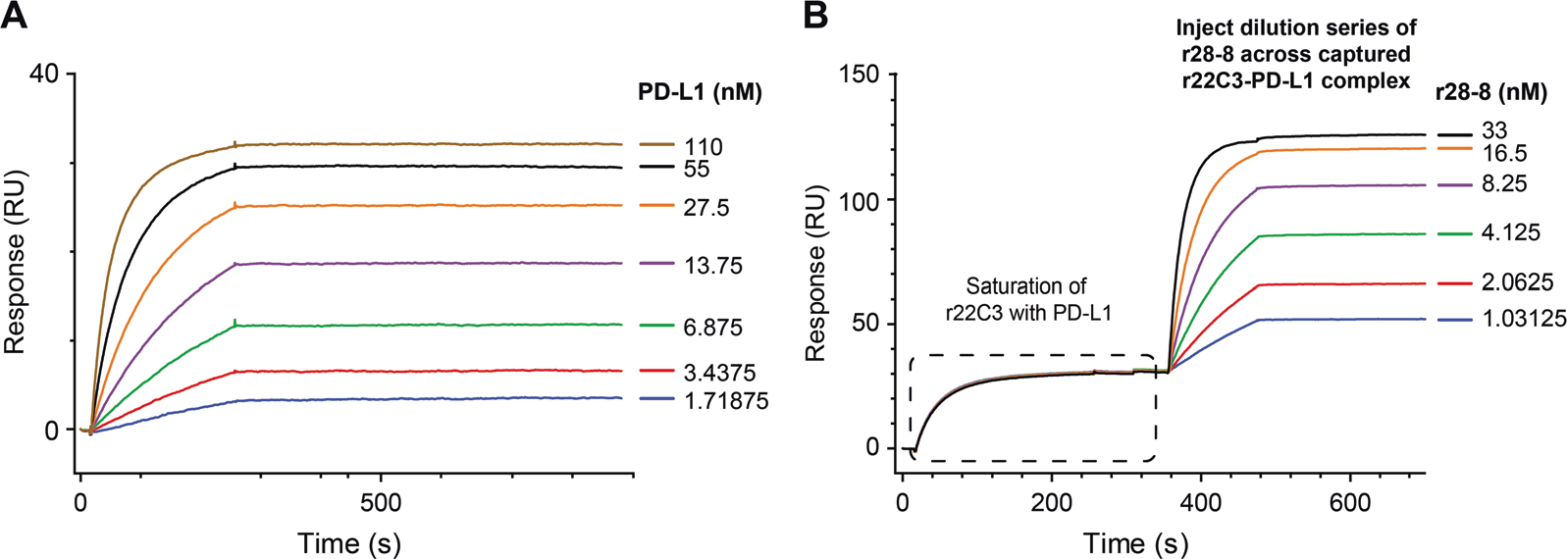
Surface Plasmon Resonance analysis of r22C3 and r28-8 antibodies to PD-L1 protein. **a** Surface Plasmon Resonance response of PD-L1 binding to 90 RU of r22C3 antibody captured on an anti-mouse IgG functionalized C1 sensor chip. **b** Saturation of r22C3 antibody with PD-L1 extracellular domain followed by injection of a dilution series of r28-8 antibody over the r22C3-PD-L1 complex, resulting in additional increase in response. Ig immunoglobulin, PD-L1 programmed cell death ligand-1, r22C3 recombinant 22C3, r28-8 recombinant 28-8, RU resonance units

SPR analysis showed potent binding between the extracellular domain of PD-L1 and r22C3 and r28-8. Titration of PD-L1 onto r22C3 captured on C1 sensor chip resulted in increased RU, yielding a dissociation constant (*K*_D_) <40 pM (Fig. 1a, Supplementary Fig. S3A). Injecting increasing concentrations of r28-8 onto the r22C3-PD-L1 complex resulted in an additional increase in RU, with no measurable dissociation during the experiment (Fig. 1b, Supplementary Fig. S3B). Both r28-8 and r22C3 antibodies had *K*_D_ < 40 pM. This observation indicates that the binding sites of r22C3 and r28-8 antibodies on PD-L1 are independent and without any significant overlap.

Confocal microscopy in HEK293 cells showed colocalization of r22C3 and r28-8 to wild-type PD-L1-green fluorescent protein (GFP)-expressing cells (Supplementary Fig. S4A–D). This result is consistent with mutually exclusive binding of r22C3 and r28-8 antibodies to distinct PD-L1 epitopes, as observed by hydrogen/deuterium exchange mass spectrometry. Antibodies r22C3 and r28-8 also colocalized to Ext4 PD-L1-GFP, a PD-L1 mutant with residues 284–290 deleted, demonstrating that these antibodies do not bind to epitopes in the cytoplasmic domain of PD-L1 (Supplementary Fig. S4E–H).

**Fig. 2:**
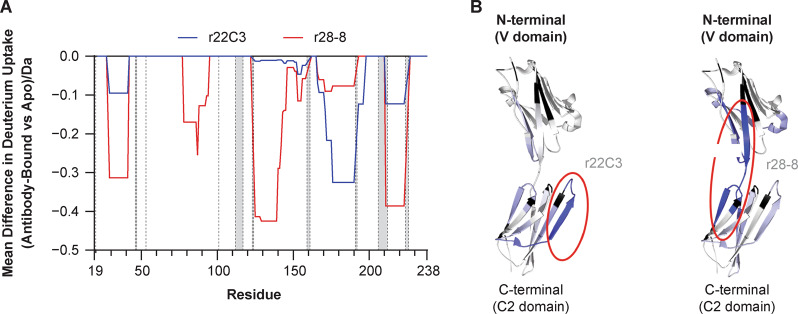
Epitope mapping of r22C3 and r28-8 anti-PD-L1 antibodies by hydrogen/deuterium exchange mass spectrometry. **a** Hydrogen/deuterium exchange mass spectrometry differential plots of antigen-binding fragments (Fab) of r22C3 and r28-8 antibodies showing mean difference in deuterium uptake between the antibody-bound state of the PD-L1 extracellular domain and the unbound (Apo) extracellular domain averaged across all time points. In the presence of antibody, residues that demonstrate differences show less deuterium uptake due to protection from exchange by the bound antibody. **b** Molecular modeling illustrating PD-L1 epitopes identified for r22C3 and r28-8 antibodies by hydrogen/deuterium exchange mass spectrometry. Epitope segments with strong and modest protection are in dark and light blue, respectively. This structural figure was generated using PyMOL with coordinates taken from PDB accession number 3FN3. Fab fragment antigen-binding, PDB Protein Data bank, PD-L1 programmed cell death ligand-1, r22C3 recombinant 22C3, r28-8 recombinant 28-8

The hydrogen/deuterium exchange performed using the extracellular domain of PD-L1 alone or in complex with r22C3 or r28-8 Fabs, induced a differential uptake of deuterium in hydrogen/deuterium exchange mass spectrometry (Fig. 2). Region 166–190 showed strong protection from deuterium exchange upon binding of r22C3 Fab; additionally, regions 28–41 and 211–223 showed marginal protection (Fig. 2). Regions 28–41, 78–94, 125–145, and 211–223 showed strong protection from deuterium exchange upon binding of r28-8, whereas region 162–196 showed marginal protection (Fig. 2). This confirmed that r28-8 and r22C3 bind different epitopes on the extracellular domain of PD-L1. Furthermore, mapping of these regions onto the structure of PD-L1 demonstrated that the predominant regions of difference between r22C3 and r28-8, identified by hydrogen/deuterium exchange mass spectrometry, lie on distinct surfaces of the PD-L1 molecule.

### Mutational analysis of PD-L1 binding by western blotting

**Fig. 3:**
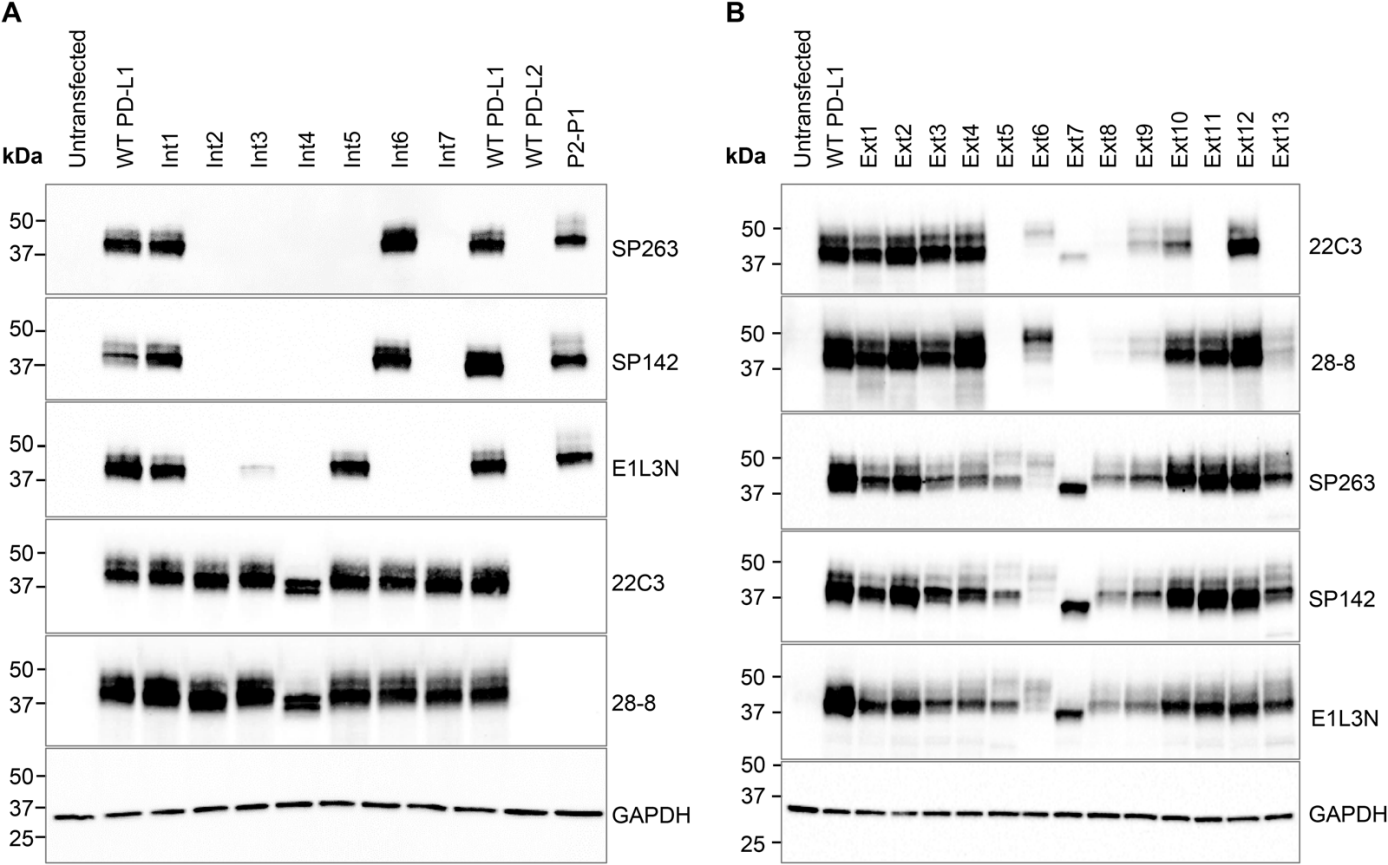
Effect of PD-L1 mutations on the binding of PD-L1 antibodies. Expi293F cells were transfected with **a** WT PD-L1, WT PD-L2, or PD-L1 constructs with mutations in the cytoplasmic domain of PD-L1 and **b** mutations in the extracellular domain of PD-L1. PD-L1 binding was detected by western blotting using SP263, SP142, E1L3N, 28-8, and 22C3 antibodies. GAPDH Glyceraldehyde 3-phosphate dehydrogenase, PD-L1 programmed cell death ligand-1, PD-L2 programmed cell death ligand-2, P2-P1, construct containing the extracellular domain of PD-L2 fused to the last 27 residues of the PD-L1 cytoplasmic domain, WT wild-type

Site-directed mutagenesis of putative epitopes was performed to obtain further detail on binding sites of the SP263, SP142, 22C3, 28-8, and E1L3N antibodies (Supplementary Table S2). Results of the effect of different mutations in the PD-L1 cytoplasmic domain on the binding of SP263, SP142, and E1L3N antibodies by western blotting are presented in Fig. 3a. Mutants Int2, Int4, and Int7 abolished SP263, SP142, and E1L3N binding. Mutant Int3 abolished SP263 and SP142 binding and reduced E1L3N binding. Mutants Int5–Int7 were designed to differentiate the binding of E1L3N from SP263/SP142 antibodies. Mutant Int5 abolished SP263 and SP142 binding without affecting E1L3N binding, while mutant Int6 abolished E1L3N binding without affecting SP263 and SP142 binding. The mutant P2-P1, containing the cytoplasmic domain of PD-L1 and the extracellular domain of PD-L2, demonstrated SP263, SP142, and E1L3N binding, confirming that these antibodies bind to the cytoplasmic domain of PD-L1. However, the P2-P1 mutant did not demonstrate 22C3 or 28-8 binding, confirming that these antibodies bind to the extracellular domain of PD-L1. All five PD-L1 antibodies tested, failed to bind the wild-type PD-L2 protein.

Western blots of PD-L1 extracellular domain mutants using 22C3 and 28-8 antibodies are presented in Fig. 3b. Mutant Ext5 abolished 28-8 and 22C3 binding. Mutant Ext6 reduced 22C3 binding and generated a shift in molecular weight of the PD-L1 protein. Mutant Ext7 abolished 28-8 binding, reduced 22C3 binding, and generated a shift in molecular weight of the PD-L1 protein. Mutants Ext8 and Ext9 reduced 28-8 and 22C3 binding. Mutant Ext11 abolished 22C3 binding but not 28-8 binding. Mutant Ext13 abolished 22C3 binding and reduced 28-8 binding. Mutants Ext7, Ext11, and Ext13 demonstrated a clear distinction in western blot binding between 28-8 and 22C3. The reduced western blot binding observed for SP263, SP142, and E1L3N with mutants Ext5, Ext6, Ext8, and Ext9 also had a numerical *H*-score reduction in the corresponding immunohistochemical mutational analysis. However, this reduction in *H*-score was minimal and in stark contrast to the total abrogation or significant reduction observed in the 28-8 and 22C3 immunohistochemical mutational analysis results. When 28-8 and 22C3 mutational analysis staining was viewed microscopically, the cell population was significantly affected, whereas SP263, SP142, and E1L3N immunohistochemical staining, despite the numerically reduced *H*-scores, exhibited almost unaltered staining intensity and proportion of stained cell population. Supplementary Fig. S5 provides the immunohistochemical mutational analysis staining data for mutant Ext5 and is representative of mutants Ext6, Ext8, and Ext9.

### Mutational analysis of PD-L1 binding by immunohistochemistry

**Fig. 4:**
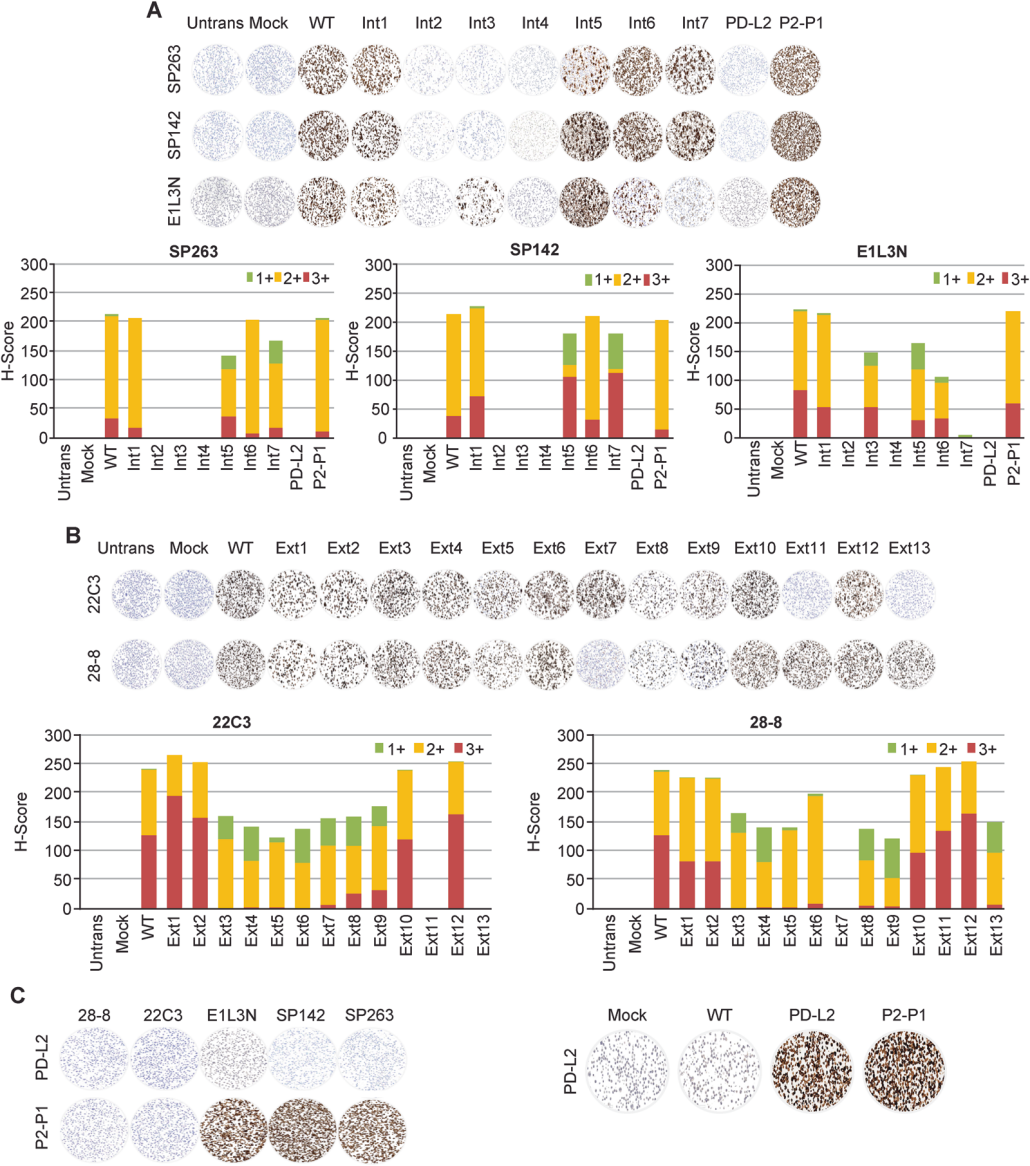
Immunohistochemical staining using PD-L1 antibodies. *H*-scores and representative images of Expi293F cell lines transfected with **a** PD-L1 WT, Int1, Int2, Int3, Int4, Int5, Int6, Int7, WT PD-L2, and PD-L2-PD-L1 fusion constructs stained with SP263 and SP142 assays and E1L3N laboratory-developed test. **b** *H*-scores and representative images of Expi293F cell lines transfected with PD-L1 WT, Ext1, Ext2, Ext3, Ext4, Ext5, Ext6, Ext7, Ext8, Ext9, Ext10, Ext11, Ext12, and Ext13 constructs stained with 22C3 and 28-8 assays. Proportion of total *H*-score contributed by cells staining at each intensity is represented by red (3+), orange (2+), and green (1+). **c** Immunohistochemical staining of PD-L2 WT and P2-P1 transfected cell lines with SP263, SP142, E1L3N, 22C3, 28-8, and PD-L2 antibodies. PD-L1 programmed cell death ligand-1, PD-L2 programmed cell death ligand-2, P2-P1 construct containing the extracellular domain of PD-L2 fused to the last 27 residues of the PD-L1 cytoplasmic domain, untrans untransfected, WT wild-type

Mutational analysis by immunohistochemistry produced a similar pattern of results to those obtained by western blotting (Fig. 4a, b, Supplementary Table S2). SP263, SP142, E1L3N, 22C3, or 28-8 antibodies demonstrated no positive staining in untransfected cells, mock transfected cells, and cells transfected with wild-type PD-L2. In contrast, all five PD-L1 antibodies demonstrated PD-L1 staining in cells transfected with wild-type PD-L1. Mutants Int2 and Int4 abolished SP263, SP142, and E1L3N staining. Mutant Int3 abolished SP263 and SP142 staining but only reduced E1L3N staining. Mutant Int5 reduced SP263, SP142, and E1L3N staining, without entirely abolishing it. Mutant Int6 reduced the proportion and intensity of E1L3N staining but had only a marginal effect on SP263 and SP142 staining. Mutant Int7 reduced the intensity of SP263 and SP142 staining and almost entirely abrogated E1L3N staining. Cells transfected with P2-P1 demonstrated strong SP263, SP142, and E1L3N staining (Fig. 4a) but were negative with 22C3 and 28-8 (Fig. 4c).

PD-L1 extracellular domain mutants Ext1, Ext2, Ext10, and Ext12 had no effect on 22C3 or 28-8 staining (Fig. 4b). Mutants Ext3–Ext6, Ext8, and Ext9 reduced the proportion and intensity of 22C3 and 28-8 staining. Mutant Ext7 reduced the proportion and intensity of 22C3 staining and completely abrogated 28-8 staining. In contrast, mutant Ext11 had no effect on 28-8 staining but completely abrogated 22C3 staining. Mutant Ext13 reduced the proportion and intensity of 28-8 staining and completely abrogated 22C3 staining. Similar to western blotting, mutants Ext7, Ext11, and Ext13 demonstrated a clear distinction between 28-8 and 22C3 staining.

### Glycosylation analysis of PD-L1

**Fig. 5:**
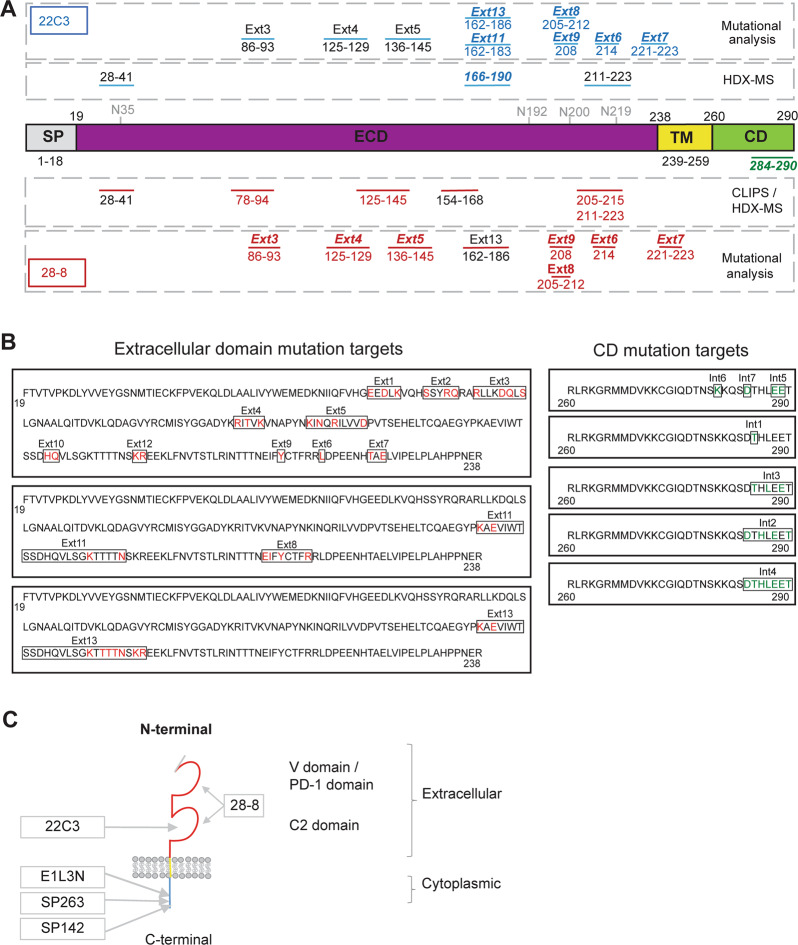
PD-L1 antibodies bind epitopes in the extracellular and cytoplasmic domains of PD-L1. **a** Illustration of putative binding epitopes of 22C3 and 28-8 PD-L1 antibodies identified by chemical linkage of peptides on scaffolds discontinuous epitope mapping, hydrogen/deuterium exchange mass spectrometry, and mutational analysis. N_35_, N_192_, N_200_, and N_219_ represent the locations of N-linked glycosylation sites. **b** Location of PD-L1 residues mutated during mutation analysis. **c** SP263 and SP142 bind identical epitopes in the cytoplasmic domain of PD-L1. E1L3N also binds an epitope in the cytoplasmic domain; however, it is different to SP263 and SP142. 22C3 and 28-8 bind epitopes on distinct surfaces of the extracellular domain of PD-L1, which are different from one another. Mutated residues are in red (ECD) and green (CD). CD cytoplasmic domain, CLIPS Chemical Linkage of Peptides onto Scaffolds, ECD extracellular domain, HDX-MS hydrogen deuterium exchange mass spectrometry, Ig immunoglobulin, PD-1 programmed cell death-1, PD-L1 programmed cell death ligand-1, SP signal peptide, TM transmembrane domain

PD-L1 contains a number of N-linked glycosylation sites at residues 35, 192, 200, and 219 (Fig. 5) [18]. Some of these sites fall within regions identified by hydrogen/deuterium exchange mass spectrometry and mutational analysis as important for binding in this study. Mutant Ext7 targets part of the N_219_ N-linked glycosylation consensus sequence (_219_NXT_221_). This mutation affects both 22C3 and 28-8 binding, abrogating 28-8 binding, reducing 22C3 binding, and generating a downward shift in molecular weight (Figs. 3b and 4b), suggesting that this region is of importance in extracellular domain-targeted antibody binding. Furthermore, enzymatic removal of N-linked glycosylation from recombinant PD-L1 also resulted in a reduction in the binding of 22C3 by western blot. Interestingly, in this context, the effect of carbohydrate removal on 28-8 binding is less apparent (Supplementary Fig. S6).

## Discussion

The results from this study demonstrated that SP263 and SP142 antibodies are indistinguishable in their recognition of a single epitope sequence (_284_DTHLEET_290_) in the cytoplasmic domain of PD-L1. Deletion of these seven residues, as demonstrated by mutant Int4, completely abolished SP263 and SP142 binding. Mutational analysis made it possible to identify residues within this sequence that are less important for binding, such as T_285_, which when mutated (mutant Int1) had no detrimental effect on SP263 and SP142 binding. In contrast, the mutation of D_284_ (mutant Int7) was observed to have a detrimental effect on immunohistochemical staining and western blot binding. Mutational analysis also demonstrated that the E1L3N antibody binds to a similar, but not identical, epitope (_280_KKQSDTHLEET_290_) in the cytoplasmic domain of PD-L1. As with SP263 and SP142, deletion of this entire region (_284_DTHLEET_290_) abolished immunohistochemical staining and western blot binding. Residues K_280_ and D_284_ were identified as being important for E1L3N binding; mutation of these residues individually resulted in drastically reduced or abrogated binding, respectively. In addition, residue T_285_ was identified as not being important for binding. These results demonstrate that the binding epitope for SP263, SP142, and E1L3N antibodies is situated within the cytoplasmic domain of PD-L1, and not in the extracellular domain as described in earlier reports [22–25]. Considering that the reported staining concordance between SP263, 22C3, and 28-8 antibodies is in marked contrast to the discordance reported for SP142, our results clearly indicate that any staining discrepancies between SP263 and SP142 cannot be epitope related as they both bind to an identical epitope.

Hydrogen/deuterium exchange mass spectrometry, SPR, and mutational analysis demonstrated that 22C3 and 28-8 antibodies recognize different discontinuous epitopes on the extracellular domain of PD-L1, which are situated on distinct surfaces of the PD-L1 molecule. The main binding epitopes for 28-8 were identified as being in regions 86–93, 125–145, and 205–223, creating a broad conformational epitope, which is influenced by glycosylation. Hydrogen/deuterium exchange mass spectrometry identified residues in the regions 78–94, 125–145, and 211–223 which are of potential importance for 28-8 binding. Mutants Ext3–Ext7 further identified that regions within these areas were important for 28-8 immunohistochemical staining and western blot binding (Fig. 5). In addition, mutants Ext8 and Ext9 and peptide arrays identified region 205–215 as encompassing residues involved in 28-8 immunohistochemical staining and western blot binding. The identification of region 154–168 by peptide array, was somewhat less convincing and may have been the result of disulfide interaction between cysteine 155 in this region and cysteine 209 in the 205–215 region. Given that this peptide was identified by peptide array using a concentration of antibody similar to that in the Dako PD-L1 IHC 28-8 pharmDx assay it is likely a genuine weaker interaction which would require further investigation. Together, all these regions create a broad conformational epitope, which is influenced by glycosylation.

The disruption of 28-8 binding by the Ext13 mutation is interesting, as this region was not identified by hydrogen/deuterium exchange mass spectrometry as being important for 28-8 binding; however, mutational analysis indicated that it does contain residues important in 28-8 binding. Mutant Ext13 shares several mutations with mutants Ext11 and Ext12, neither of which appear to affect 28-8 binding. Therefore, it is possible to infer that the residues in mutant Ext13, not mutated in mutants Ext12 and Ext11 (residues 180–182), may be important in 28-8 binding. Alternatively, it is possible that structural disruption, due to the extent of mutations within mutant Ext13, may have inhibited 28-8 binding.

The main binding epitopes for 22C3 were identified in the regions 162–183, with an additional contribution from region 205–223. Hydrogen/deuterium exchange mass spectrometry identified that the predominant region for 22C3 binding encompassed the residues 166–190 and mutational analysis, using mutants Ext11 and Ext13, identified that residues 162–183 are important for 22C3 immunohistochemical staining and western blot binding. The 22C3 epitope identified was similar to that reported in the 22C3 patent (residues 156–178) [27]. In addition, hydrogen/deuterium exchange mass spectrometry identified some weaker binding contributions from regions 28–41 and 211–223; these regions encompassed residues, within the Ext6–Ext8 mutations, and mutational analysis identified that these residues are important for 22C3 immunohistochemical staining and western blot binding. Mutational analysis, using mutants Ext5, Ext8, and Ext9 identified that these mutations demonstrated abrogation or reduction of 22C3 immunohistochemistry staining and western blot binding. Further, these residues could not be identified using peptide arrays. Mutational analysis, using mutants Ext3 and Ext4 also demonstrated a reduction in 22C3 immunohistochemical staining. Despite the extracellular domain antibodies, 22C3 and 28-8, sharing some common binding regions, hydrogen/deuterium exchange mass spectrometry identified one significant factor that clearly differentiated them, namely that the predominant binding regions lie on distinct surfaces of the PD-L1 molecule. Hydrogen/deuterium exchange mass spectrometry also identified a potential contribution of varying importance to 28-8 and 22C3 binding in region 28–41. The PD-L1 protein has an N-linked glycosylation site situated at residue 35 in this region. Similar N-linked glycosylation sites are also located at residues 192, 200, and 219 [18, 39]. It is worth noting, that hydrogen/deuterium exchange mass spectrometry identified a similar contribution to 28-8 and 22C3 binding in region 211–223. Mutation Ext7 partially covers this region, overlapping T_221_, which is part of the N-linked glycosylation consensus sequence (_219_NXT_221_). Mutational analysis, using mutant Ext7, demonstrated its importance in 22C3 and 28-8 binding. Immunohistochemistry staining and western blot binding was reduced for 22C3 and abrogated for 28-8, additionally there was a reduction in PD-L1 protein molecular weight observed with both antibodies (Figs. 3b and 4b). This highlights that this is a region of significance in extracellular domain-targeted antibody binding. Removal of these N-linked oligosaccharide groups, using PNGase F, resulted in decreased detection of recombinant PD-L1 by western blotting, for the 22C3 antibody (Supplementary Fig. S6). This suggests that glycosylation plays a role, of varying importance, in 28-8 and 22C3 target binding, depending upon experimental context. The immunohistochemistry results for Ext7 and the varied effects seen in western blotting indicate the need for further investigation of the effects of glycosylation on the binding of both these antibodies in patient-derived samples.

A combination of approaches, including peptide arrays epitope mapping, hydrogen/deuterium exchange mass spectrometry, and mutational analysis, was used to identify all possible epitopes important for binding. Chemical linkage of peptides on scaffolds peptide array epitope mapping identifies linear, discontinuous, and conformational epitopes by converting the target protein into an extensive library of conformationally constrained peptide mimics, which have sequences that are not adjacent in the primary sequence brought together by a scaffold [36]. Hydrogen/deuterium exchange mass spectrometry identifies protein conformation in solution. Unlike peptide arrays, it can detect changes induced by binding events in regions that are distant from the epitope, a phenomenon resulting from allosteric effects [40]. Western blotting utilized reduced and denatured samples, whereby secondary and tertiary protein structures were disrupted resulting in unfolding and effective linearization of proteins; hence, antibodies could predominantly detect linear portions of epitopes. Alternatively, formalin fixation stabilizes tissue morphology and secondary and tertiary protein structure, so that antibodies can detect proteins in the “formalin-fixed paraffin-embedded state.” [41, 42] Commercially available PD-L1 assay antibodies are generated to specifically recognize epitopes present in formalin-fixed paraffin-embedded tissue sections, where the protein structure can be uniquely disrupted in comparison with its native form. The binding of these antibodies to formalin-fixed paraffin-embedded protein structure in immunohistochemistry assay protocols can differ from the more linear protein structures created under western blotting conditions. In this study, the performance of the PD-L1 antibodies in their respective immunohistochemistry assay protocols is of far greater significance, as any reduction in PD-L1 staining would have a detrimental effect on patient status in the clinical setting.

Despite the PD-L1 assay antibodies exhibiting variation in epitope binding, studies have indicated that a statistically significant staining concordance exists between 22C3, 28-8, and SP263 antibodies, with only SP142 generating discordant immunohistochemical staining [28, 34]. The discordance between SP142 and SP263 assays, despite binding to an identical epitope, is most likely a result of minor differences in assay protocol design. When SP142 is incorporated in alternative assay protocols, it will demonstrate staining concordant with that of SP263 and the other antibodies [14, 43], clearly confirming that the SP142 antibody has no intrinsic binding issues (Supplementary Fig. S7).

A limitation of this study remains that despite different techniques used, no single technique could unequivocally determine the binding for all epitopes. Variation in the expression levels of some of the PD-L1 mutants may have confounded the identification of some of the (discontinuous) binding epitopes. Signals from the glycosylation moieties on PD-L1 may also have affected the identification of discontinuous epitopes by hydrogen/deuterium exchange mass spectrometry and should be explored further to determine their role in 22C3/28-8 antibody binding. Minor reductions noted in the immunohistochemical staining expression data are a result of the rationale behind the semiquantitative *H*-scoring approach; placing a small proportion of PD-L1 stained cells into a lower or higher intensity category can make a numerical impact. In immunohistochemical mutational analysis, staining in cell cores displaying minor numerical *H*-score reductions, microscopically exhibited virtually unaltered immunohistochemical staining intensity and proportion of PD-L1 stained populations. This is in stark contrast to the cell cores displaying major numerical *H*-score reductions; microscopically they exhibit clear unambiguous abrogation or significant reductions, with all or most of the cell population affected (Supplementary Fig. S5). The study strengths include performing epitope mapping with a high degree of rigor using highly sensitive current technologies. Results were validated using clinically approved PD-L1 immunohistochemistry assays, performed in accordance with their product inserts, to confirm identification of epitope sequences in cell lines expressing various epitope mutants.

This study confirms the antibody-binding epitope data provided by PD-L1 diagnostic companies and further expands the understanding of these epitope binding regions. The apparent difference observed between the performance of the SP142 assay and the other assays, has been shown to be more likely attributable to assay design and protocol variables than to epitope binding. Based on data from this study and published PD-L1 assay concordance data [14, 28, 29, 34], we would suggest that any differences observed in the clinical performance of PD-1 and PD-L1 therapeutics are highly unlikely to be attributable to differences in the epitopes recognized by their associated PD-L1 immunohistochemistry assay antibodies. It is more appropriate to suggest that they may arise from differences in the choice of cutoff/algorithm/clinical trial design, properties intrinsic to the antibodies, or the therapies themselves.

## Supplementary information

Supplementary material
