# Correction: Mapping the binding sites of antibodies utilized in programmed cell death ligand-1 predictive immunohistochemical assays for use with immuno-oncology therapies

**DOI:** 10.1038/s41379-019-0379-5

**Published:** 2019-10-22

**Authors:** Nicola L. Lawson, Carly I. Dix, Paul W. Scorer, Christopher J. Stubbs, Edmond Wong, Liam Hutchinson, Eileen J. McCall, Marianne Schimpl, Emma DeVries, Jill Walker, Gareth H. Williams, James Hunt, Craig Barker

**Affiliations:** 1 Precision Medicine, Oncology R&D, AstraZeneca, Cambridge, UK; 2 Antibody Discovery and Protein Engineering (ADPE), R&D, AstraZeneca, Cambridge, UK; 3 Structure, Biophysics and Fragment-Based Lead Generation, Discovery Sciences, BioPharmaceuticals R&D, AstraZeneca, Cambridge, UK; 4 Spirogen, AstraZeneca, London, UK; 5 FM Operations, BioPharmaceuticals R&D, AstraZeneca, Cambridge, UK; 6 Precision Medicine, Oncology R&D, AstraZeneca, Cambridge, UK; 7 Oncologica UK Ltd, Cambridge, UK

**Keywords:** Predictive markers, Cancer immunotherapy

**Correction to: Modern Pathology**

10.1038/s41379-019-0372-z

This article was erroneously published with standard licence and copyright, but it is in fact published with open access, so has a CC BY licence and the copyright line is ‘The Author(s)’. This has been updated in the original article.

