# Supplementary material for: Mapping the binding sites of antibodies utilized in programmed cell death ligand-1 predictive immunohistochemical assays for use with immuno-oncology therapies
Source: Mod Pathol. 2019 Sep 26;33(4):518–30. doi: 10.1038/s41379-019-0372-z (PMC8075905; doi:10.1038/s41379-019-0372-z)

# **Supplementary appendix**

1. **Recombinant 22C3 and 28-8 antibody production, purification, and fragment antigen-binding (Fab) generation**

Chinese Hamster Ovary transient expression system engineered to express the Epstein-Barr virus nuclear antigen-1 with co-expression of the glutamine synthetase gene (CHO-EBNA-GS) cells, cultured in CD CHO media (10743029, Gibco, USA), were co-transfected with either the 28-8 variable heavy-chain (pEU33.0) and variable light-chain (pEU34.0) constructs or the 22C3 variable heavy-chain (pEU23.2) and variable light-chain (pEU22.2) constructs using PEI MAX (24765, Polysciences, Inc., USA), as described earlier.^32^ Transfected cells were cultured in roller bottles at 37℃, 140 rpm, and with 8% CO_2_. Samples were quantified for cell density and cell viability on days 1, 4, and 14 post-transfection and supernatant was harvested on day 14. Protein expressed in cell culture supernatant was detected by sodium dodecyl sulfate polyacrylamide gel electrophoresis (SDS-PAGE).

Monoclonal recombinant 28-8 (r28-8) and recombinant 22C3 (r22C3) antibodies were purified from the CHO-EBNA-GS cell culture supernatant by protein A and protein G affinity chromatography, respectively, using an AKTAxpress system (GE Healthcare Life Sciences, Sweden). Purified antibodies were dialyzed in phosphate-buffered saline (PBS; 137 mM NaCl, 2.7 mM KCl, 8 mM Na_2_HPO_4_, and 2 mM KH_2_PO_4_). Briefly, culture supernatant containing the antibodies were loaded onto either the protein A column (5 mL, HiTrap Protein A HP, GE healthcare Life Sciences, Sweden) or protein G column (5 mL, HiTrap Protein G HP, GE healthcare Life Sciences, Sweden). The columns were washed with at least 10 column volumes of PBS before eluting the antibodies with 100 mM Glycine-HCl (pH 2.7) into 900 μL fractions. The antibodies were neutralized with 100 μL of 1M Tris-HCl (pH 7.4) for each fraction collected. Fractions containing the antibody of interest were pooled, dialyzed in PBS, and concentrated to 1 mg/mL. To confirm the molecular weight, purified antibody (5μg) was dissolved in sample buffer (69.45 mM Tris-HCl [pH 6.8], 11.1% glycerol, 1.1% SDS, 0.005% bromophenol blue) with 2-mercaptoethanol to a final concentration of 0.355 M (reducing condition) or without (non-reducing condition) and analyzed by SDS-PAGE (NuPage 10% Bis-Tris gel) at 220 V for 36 min in 1× MOPS buffer and stained with InstantBlue (ISB1L; Sigma-Aldrich, UK). Protein bands were excised and subjected to mass spectrometry to confirm peptide sequence. Protein bands were reduced with 10 mM dithiothreitol/100 mM ammonium bicarbonate at 65°C for 45 min. Protein bands were alkylated using 50 mM iodoacetamide/100 mM ammonium bicarbonate and incubating the bands at room temperature in the dark for 20 min. The bands were digested with 20 μL of 10 ng/μL trypsin and chymotrypsin overnight at 37°C. The resultant digests were loaded on the NanoAcquity LC coupled to the Synapt G2 mass spectrometer. Data were searched using the BioPharmaView software and manually verified.

Fab fragments of r28-8 and r22C3 antibodies were generated by proteolytic cleavage. r22C3 was concentrated to 15 mg/mL and incubated with 0.5 mL of agarose-immobilized ficin (44881, Thermo Scientific, USA) for 7 h at 37℃. r28-8 was concentrated to 1.15 mg/mL and incubated with 2 mL of agarose-immobilized papain (20341, Thermo Scientific, USA) for 24 h at 37℃. Fragment crystallizable (Fc) fragments were removed by affinity chromatography on a HiTrap protein A High Performance 1 mL pre-packed column (17‑0402-01, GE Healthcare, Sweden) as per the manufacturer’s protocol. The flow-through containing the Fab fragments was concentrated and subjected to size exclusion chromatography on a Superdex 200 10/300 GL column (17-5175-01, GE Healthcare, USA) with isocratic elution in 2× Dulbecco's PBS containing 5.33 mM KCl, 2.94 mM KH_2_PO4, 16.12 mM Na_2_HPO4, 275.86 mM NaCl (14200-067, Gibco, USA). Fractions were collected, pooled, and concentrated to final concentrations of 4.71 mg/mL for r22C3 Fab and 2.34 mg/mL for r28-8 Fab in 2× Dulbecco's PBS buffer and subjected to mass spectrometry to verify molecular weight.

1. **Immunofluorescence microscopy**

HEK293 cells (American Type Culture Collection) were cultured at a density of 1.2 × 10^4^ per well in 96-well CellCarrier Ultra Microplates (6055302; Perkin Elmer, USA) and incubated at 37°C and 5% CO_2_. At 24 h post-seeding and 40–80% confluency, cells were transfected with carboxy-terminal green fluorescent protein (GFP)-tagged programmed cell death ligand-1 (PD-L1) wild-type or mutant constructs using Fugene HD (E2311; Promega, USA) at a ratio of 1 μg DNA to 3 μL Fugene, as per manufacturer’s instructions. At 48 h post-transfection, media was removed. Cells were washed in PBS (pH 7.4; 137 mM NaCl, 2.7 mM KCl, 8 mM Na_2_HPO_4_, and 2 mM KH_2_PO_4_) and fixed for 10 min in 4% paraformaldehyde. Cells were blocked in 1.1% bovine serum albumin for 30 min and incubated with r22C3 (1:1000) or r28-8 (1:1000) antibodies (1 mg/mL) for 1 h at room temperature. Cells were washed with PBS, incubated with goat anti-mouse Alexa Fluor 647 (r22C3; A21235) or goat anti-rabbit Alexa Fluor 568 (r28-8; A11011) conjugated secondary antibodies (1:500; Thermo Fisher Scientific, USA) in blocking buffer (5% non-fat milk in Tris-buffered saline [20 mM Tris and 150 mM NaCl] containing 0.05% Tween-20 [TBST]) containing Hoecsht dye (1:10000; H3570, Invitrogen, USA) for 1 h, and washed again with PBS. For dual staining, cells were incubated with a second primary antibody (1:1000; r22C3 or r28-8 [1 mg/mL]) for 1 h at room temperature, washed, incubated with appropriate Alexa dye conjugated secondary antibody, and washed again. Images were obtained by fluorescence microscopy on CV7000 confocal microscope (Yokogawa, Japan) using a 60× water immersion objective lens.

1. **Expression of PD-L1 mutants in Expi293F cells**

Expi293F cells at a density of 3–3.4 × 10^6^ cells/mL in a final volume of 10 mL were transiently transfected with 15 μg mutant PD-L1 or programmed cell death ligand-2 (PD-L2) construct and 60 μL of 1 mg/mL PEI Max transfection reagent (24765, Polysciences, Inc., USA) in a 1:4 ratio, and cells were cultured in 50 mL tubespins at 37°C with 8% CO_2_ for 48 h. Cells were harvested for lysate or cell core production by centrifugation. For lysate production, cells were washed twice in ice-cold PBS. Cell pellets were resuspended in ice-cold radioimmunoprecipitation assay lysis buffer (25 mM Tris-HCl [pH 7.6], 150 mM NaCl, 1% NP-40, 1% sodium deoxycholate, 0.1% SDS) supplemented with a protease and phosphatase inhibitor cocktail (88669, Thermo Scientific, USA). Cell lysates were incubated on ice for 30 min before being transferred to a microfuge and centrifuged at 13000 rpm for 15 min. Supernatant was removed and analyzed using SDS-PAGE and western blotting.

1. **SDS-PAGE and western blotting**

Total protein in cell lysates was quantified by bicinchoninic acid assay (BCA; 23225, Thermo Scientific, USA) using a pre-diluted bovine serum albumin standard curve (23208, Thermo Scientific, USA). Samples were equalized, mixed with 4× Laemmli Buffer (1610747, Bio-Rad, USA) containing 2-mercaptoethanol, and denatured by boiling at 90°C for 10 min. Proteins were separated on a 4–15% Tris-glycine gel by SDS PAGE (5671084, Bio-Rad, USA) and transferred to nitrocellulose membrane (1620115, Bio-Rad, USA) for 60 min at 100 V. Membranes were blocked in blocking buffer overnight at 4°C or for 1 h at room temperature. Blocked membranes were incubated for 1 h at room temperature with primary antibody in blocking buffer at the following dilutions: SP263 (1:250; 790-4905, Ventana Medical Systems, Inc., USA), SP142 (1:1000; M4422, Spring BioScience, USA), 28-8 (1:1000; ab205921, Abcam, UK), 22C3 (1:1000; M365329, Dako, Denmark), r22C3 (1:1000), r28-8 (1:1000); and from Cell Signaling Technologies, Inc. (USA), E1L3N (1:1000; 13684), PD-L2 (1:1000; clone D7U8C [82723]), and GAPDH (1:1000; clone 14C10 [2118]). Membranes were washed three times for 5 min each in TBST, then incubated with anti-rabbit IgG (1:3000; 7074S) or anti-mouse IgG (1:3000; 7076S) HRP-conjugated secondary antibodies (Cell Signaling Technologies, Inc., USA) at room temperature for 1 h. Following three further washes with TBST, blots were visualized with chemiluminescent HRP substrate (WBLUR0500, Millipore, USA) using the ChemiDoc MP imaging system (Bio-Rad, USA).

1. **Mass spectrometry of deglycosylated PD-L1**

A total of 0.75 µg of protein was injected onto the ExionLC and separated using a Waters XBridge Protein BEH C4 column (300 Å, 3.5 μm, 2.1 mm × 50 mm, Waters USA) at acid in acetonitrile) were used with a total run time of 5 min at a moving flow rate of 0.2–0.5 mL/min. Acquisition 80°C. Standard mobile phases (mobile phase A: 0.1% formic acid in water, mobile phase B: 0.1% formic) was performed on X500B QTOF with a Turbo V™ ion source using protein mode acquisition and detector voltage selected over a range of 500–3000 Da. Electrospray parameters were as follows: curtain gas, 50; ion source gas 1, 50 psi; ion source gas 2, 50 psi; and temperature, 400°C.

For peptide mass fingerprinting, the protein samples were run on NuPAGE 10% Bis-Tris gel at 200 V for 50 min in 2-(N-morpholino)ethanesulfonic acid (MES) buffer and stained with InstantBlue protein stain. Afterwards, the protein bands were excised and washed with the following solutions: 180 μl of H_2_O for 10 min, then 180 μl of 50% acetonitrile/H_2_O solution for 10 min, then 180 μl of 100 mM ammonium bicarbonate for 10 min, and then 180 μl of 50 mM ammonium bicarbonate/50% acetonitrile solution for 10 min. The bands were then incubated with 75 μl of 10 mM dithiothreitol/100 mM ammonium bicarbonate solution and incubated at 65°C for 45 min before discarding the solution. The bands were incubated with 75 μl of 50 mM iodoacetamide/100 mM ammonium bicarbonate solutions at room temperature in the dark for 20 min. After discarding the solution, the bands were washed with the following solutions: 180 μl of 50 mM ammonium bicarbonate for 10 min, then 180 μl of 50 mM ammonium bicarbonate/50% acetonitrile solutions for 10 min, and then 180 μl of acetonitrile for 10 min. The bands were dried down in a vacuum dryer for 10 min. A 20 μl solution of trypsin (10 ng/μl prepared in 50 mM ammonium bicarbonate, pH 8.0) was added to each sample. Protein bands were left for 10–15 min, then topped up with 5 μl of 50 mM ammonium bicarbonate solution to prevent the pieces from drying out. Samples were incubated overnight at 37°C. A 20 μl solution of 0.1% trifluoroacetic acid/60% acetonitrile was added to the digested gel pieces, and the pieces were placed on a shaker for 10 min; this step was repeated three times. The supernatant was extracted and pooled in a second plate. The samples were dried down for 15 min in a vacuum dryer. The sample volumes were made up to 30 μl with 0.1% trifluoroacetic acid.

For electrospray ionization MS/MS using X500B mass spectrometer (SCIEX), 10 μl of the sample was subjected to chromatography using the ExionLC™ chromatography system (SCIEX) and the outlet flow run directly into the X500B for analysis at a flow rate of 300 μl/min. A 20 min reversed phase gradient was run using an Acquity UPLC CSH C18 analytical column (2.1 × 100 mm; Waters, USA). The X500B collected data in positive ion mode and an auto switching setup was initiated with automatic precursor selection based on peak intensity and charge state. The collision energies were automatically adjusted based on the precursor. Nitrogen was used as the collision gas.

The subsequent data files generated were searched against the in house or SWISS-PROT database using the MASCOT Daemon software. The searches were then manually verified.

1. **SP142 laboratory developed test (LDT) assay protocol**

CD274 reference standard cell line slides (HDX24013, Horizon Discovery, Cambridge, UK) consisting of four genetically engineered CD274 protein expressing cell line cores, supplied as 4 µm formalin-fixed, paraffin-embedded (FFPE) sections, were stained with Dako PD-L1 IHC 22C3 PharmDx assay and Dako PD-L1 IHC 28-8 PharmDx assay on automated Dako Link 48 autostainer and with VENTANA PD-L1 (SP263) Assay and VENTANA PD-L1 (SP142) Assay on Ventana Benchmark Ultra, according to manufacturer’s instructions at Hematogenix (Tinley park, IL, USA).

CD274 reference standard cell line slides were stained with an SP142 LDT assay on a Leica Bond RX automated staining instrument using the Bond Polymer Refine Detection System IHC protocol F (DS9800, Leica Biosystems, Germany) with the following modifications: 30-min heat-induced epitope retrieval using Bond™ Epitope Retrieval Solution 2 (AR9640); 20-min Leica Protein Block (RE7102), 30-min SP142 antibody (M4422; Spring BioScience, USA) incubation at 1/200 dilution in BOND Antibody Diluent (AR9352); 15 min Bond Refine Polymer, 10 s DAB Enhancer BOND (AR9432); 2 min 30 s Bond Refine Hematoxylin (DS9800). Following immunohistochemistry (IHC) staining, slides were dehydrated through graded alcohols, coverslipped, and mounted with ClearVue XYL mounting medium (4212, Thermo Scientific, USA). Tonsil and placenta tissues were used as positive controls and untransfected FFPE Expi293F cells served as negative control. Stained slides were digitally scanned using the Aperio ScanScope AT2 slide scanner (Leica Biosystems, Germany), and visualized using ImageScope software.

# **Supplementary Tables**

## **Supplementary table S1**. Sequences of 28-8 and 22C3 recombinant antibodies

| **Rabbit r28-8** | **Heavy chain:**  QCLSVEESGGRLVTPGTPLTLTCTASGFTITNYHMFWVRQAPGKGLEWIGVITSSGIGSSSTTYYATWAKGRFTISKTSTTVNLRITSPTTEDTATYFCARDYFTNTYYALDIWGPGTLVTVSSGSFKAPSVFPLAPCCGDTPSSTVTLGCLVKGYLPEPVTVTWNSGTLTNGVRTFPSVRQSSGLYSLSSVVSVTSSSQPVTCNVAHPATNTKVDKTVAPSTCSKPTCPPPELLGGPSVFIFPPKPKDTLMISRTPEVTCVVVDVSQDDPEVQFTWYINNEQVRTARPPLREQQFNSTIRVVSTLPIAHQDWLRGKEFKCKVHNKALPAPIEKTISKARGQPLEPKVYTMGPPREELSSRSVSLTCMINGFYPSDISVEWEKNGKAEDNYKTTPAVLDSDGSYFLYSKLSVPTSEWQRGDVFTCSVMHEALHNHYTQKSISRSPGK  **Light chain:**  ALVMTQTPSSTSTAVGGTVTIKCQASQSISVYLAWYQQKPGQPPKLLIYSASTLASGVPSRFKGSRSGTEYTLTISGVQREDAATYYCLGSAGSFGGGTEVVVKDPVAPTVLIFPPAADQVATGTVTIVCVANKYFPDVTVTWEVDGTTQTTGIENSKTPQNSADCTYNLSSTLTLTSTQYNSHKEYTCKVTQGTTSVVQSFNRGD |
| --- | --- |
| **Mouse r22C3** | **Heavy chain:**  QVHLQQSGAELAKPGASVKMSCKASGYTFTSYWIHWIKQRPGQGLEWIGYINPSSGYHEYNQKFIDKATLTADRSSSTAYMHLTSLTSEDSAVYYCARSGWLIHGDYYFDFWGQGTTLVTVSSAKTTPPSVYPLAPGSAAQTNSMVTLGCLVKGYFPEPVTVTWNSGSLSSGVHTFPAVLQSDLYTLSSSVTVPSSTWPSETVTCNVAHPASSTKVDKKIVPRDCGCKPCICTVPEVSSVFIFPPKPKDVLTITLTPKVTCVVVDISKDDPEVQFSWFVDDVEVHTAQTQPREEQFNSTFRSVSELPIMHQDWLNGKEFKCRVNSAAFPAPIEKTISKTKGRPKAPQVYTIPPPKEQMAKDKVSLTCMITDFFPEDITVEWQWNGQPAENYKNTQPIMDTDGSYFVYSKLNVQKSNWEAGNTFTCSVLHEGLHNHHTEKSLSHSPGK  **Light chain:**  DIVMSQSPSSLAVSAGEKVTMTCKSSQSLLHTSTRKNYLAWYQQKPGQSPKLLIYWASTRESGVPDRFTGSGSGTDFTLTISSVQAEDLAVYYCKQSYDVVTFGAGTKLEIKGADAAPTVSIFPPSSEQLTSGGASVVCFLNNFYPKDINVKWKIDGSERQNGVLNSWTDQDSKDSTYSMSSTLTLTKDEYERHNSYTCEATHKTSTSPIVKSFNRNEC |

## **Supplementary table S2**. Sequences of PD-L1 mutants. Mutated residues are in red

| **Construct** | **Sequence** | **Construct** | **Sequence** |
| --- | --- | --- | --- |
| WT PD-L1 | _70_GEEDLKVQHSSYRQRARLLKDQLSLG_95_ | WT PD-L1 | _162_KAEVIWTSSDHQVLSGKTTTTNSKR_186_ |
| Ext1 | _70_GAEALAVQHSSYRQRARLLKDQLSLG_95_ | Ext10 | _162_KAEVIWTSSDAAVLSGKTTTTNSKR_186_ |
| Ext2 | _70_GEEDLKVQHASYAARARLLKDQLSLG_95_ | Ext11 | _162_AAAVIWTSSDHQVLSGATTTTASKR_186_ |
| Ext3 | _70_GEEDLKVQHSSYRQRAALLKAALALG_95_ | Ext12 | _162_KAEVIWTSSDHQVLSGKTTTTNSAA_186_ |
| WT | _125_RITVKVNAPYNKINQRILVVD_145_ | Ext13 | _162_AAAVIWTSSDHQVLSGATDAATSAA_186_ |
| Ext4 | _125_AIAVAVNAPYNKINQRILVVD_145_ | WT PD-L1 | _280_KKQSDTHLEET_290_ |
| Ext5 | _125_RITVKVNAPYNAIAQAILVVA_145_ | Int1 | _280_KKQSDAHLEET_290_ |
| WT PD-L1 | _205_EIFYCTFRRLDPEENHTAE_223_ | Int2 | _280_KKQSATALAEA_290_ |
| Ext6 | _205_EIFYCTFRRYDPEENHTAE_223_ | Int3 | _280_KKQSDAHAEAT_290_ |
| Ext7 | _205_EIFYCTFRRLDPEENHAAA_223_ | Int4 | _280_KKQS_283_ |
| Ext8 | _205_AAFACTFARLDPEENHTAE_223_ | Int5 | _280_KKQSDTHLAAT_290_ |
| Ext9 | _205_EIFACTFRRLDPEENHTAE_223_ | Int6 | _280_AKQSDTHLEET_290_ |
|  |  | Int7 | _280_KKQSATHLEET_290_ |

PD-L1, programmed cell death ligand-1; WT, wild-type.

# **Supplementary Figures**

## **Supplementary figure S1**. Verification of r22C3 and r28-8 antibodies by SDS-PAGE. (A) 1 μg purified r22C3, and (B) 1 μg of 28-8 (Abcam) and r28-8 antibodies in reducing (4× Laemmli buffer *plus β-mercaptoethanol to a final concentration of 5% or 710 mM) and non-reducing (4× Laemmli buffer) conditions were run on a NuPAGE 10% Bis-Tris gel, at 200 V for 50 min in MES buffer and stained with InstantBlue protein stain. MES, 2-(N-morpholino) ethanesulfonic acid; r22C3, recombinant 22C3; r28-8, recombinant 28-8; SDS-PAGE, sodium dodecyl sulfate-polyacrylamide gel electrophoresis


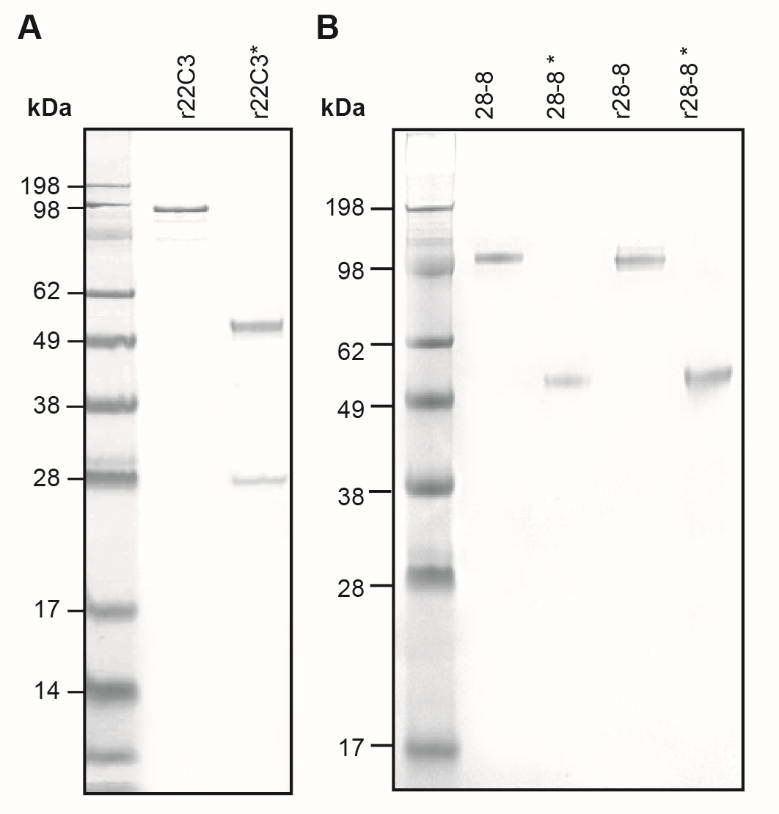


## **Supplementary figure S2.** Putative epitopes identified by linear and discontinuous epitope mapping for PD-L1 antibodies. Ig, immunoglobulin; PD-L1, programmed cell death ligand-1; TM, transmembrane

##
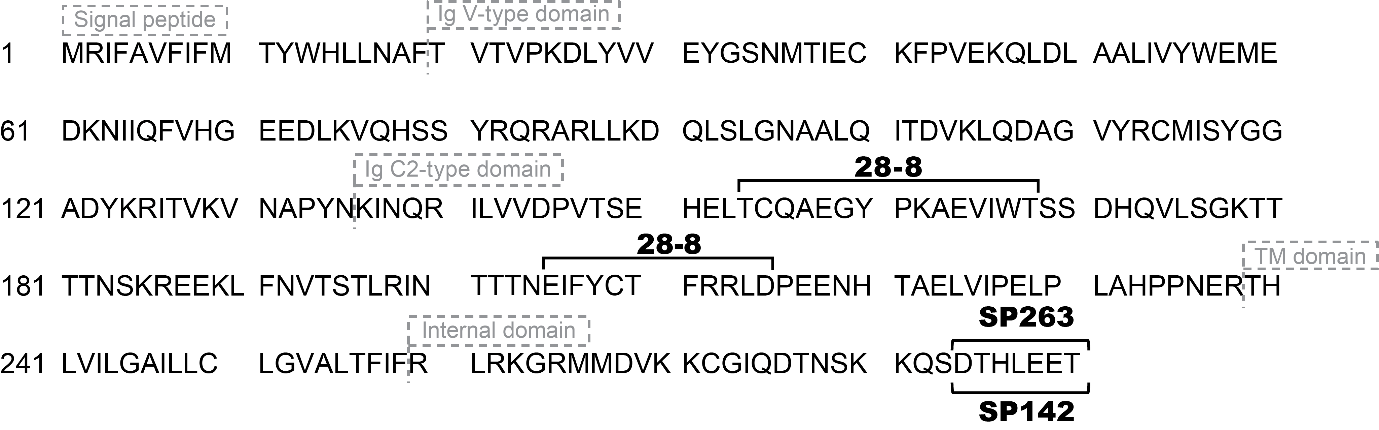


## **Supplementary figure S3**. Affinity/kinetics of interactions between r22C3 and r28-8 antibodies and the PD-L1 extracellular domain. (A) PD-L1 binding to immobilized r22C3 and (B) r28-8 binding to PD-L1 captured on r22C3 data were fit using a 1:1 interaction model; colored lines are the experimental data and the black lines represent the fits. K_D_, dissociation constant; k_off_, dissociation rate constant; k_on_, association rate constant; PD-L1, programmed cell death ligand-1; r22C3, recombinant 22C3; r28-8, recombinant 28-8. RU, resonance units.


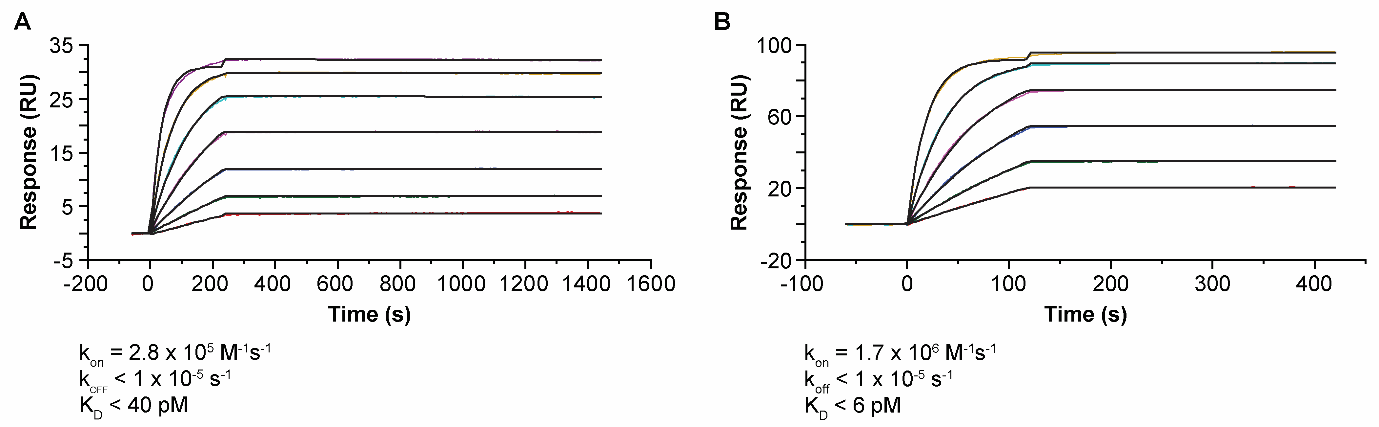


## **Supplementary figure S4**. Confocal microscopy demonstrating simultaneous binding of r22C3 and r28-8 to PD-L1 due to distinct binding epitopes. HEK293 cells were transfected with WT PD-L1-GFP construct (A–D) or Ext4 PD-L1-GFP construct (E–H) stained sequentially with r22C3 and r28-8 antibodies and imaged for GFP (A, E), r22C3 and r28-8 combined (B, F), r22C3 (C, G), and r28-8 (D, H). The ability of r22C3 and r28-8 antibodies to bind to the expressed Ext4 PD-L1-GFP protein, which contains a deletion of residues 284–290, demonstrates that these antibodies do not bind to epitopes in this C-terminal region. GFP, green fluorescent protein; PD-L1, programmed cell death ligand-1; r22C3, recombinant 22C3; r28-8, recombinant 28-8.


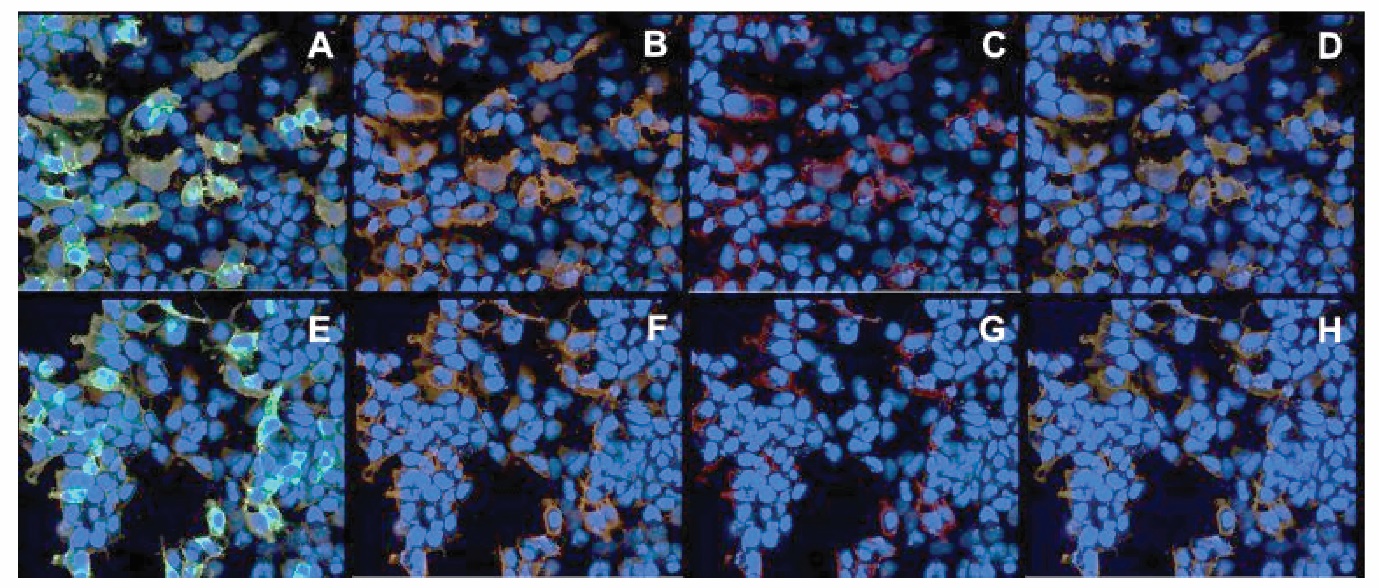


## **Supplementary figure S5**. Difference in IHC staining appearance between significant H-score reductions and minor H-score reductions using IHC mutational analysis with mutant Ext5. H-scores and representative images of Expi293F cell lines transfected with WT PD-L1 and mutant Ext5 constructs and stained with SP263, SP142, E1L3N LDT, 22C3 and 28-8 assays are presented. Despite, numerically reduced H-scores, microscopic analysis showed that IHC staining intensity and proportion of stained cell population was almost unaltered with cytoplasmic domain PD-L1 antibodies SP263, SP142 and E1L3N. In contrast, extracellular domain PD-L1 antibodies 28-8 and 22C3 clearly demonstrated a significant change in staining intensities and positive cell population. Proportion of total H-score contributed by cells staining at each intensity is represented by red (3+), orange (2+), and green (1+). IHC, immunohistochemical; LDT, laboratory developed test; PD-L1, programmed cell death ligand-1; WT, wild type.


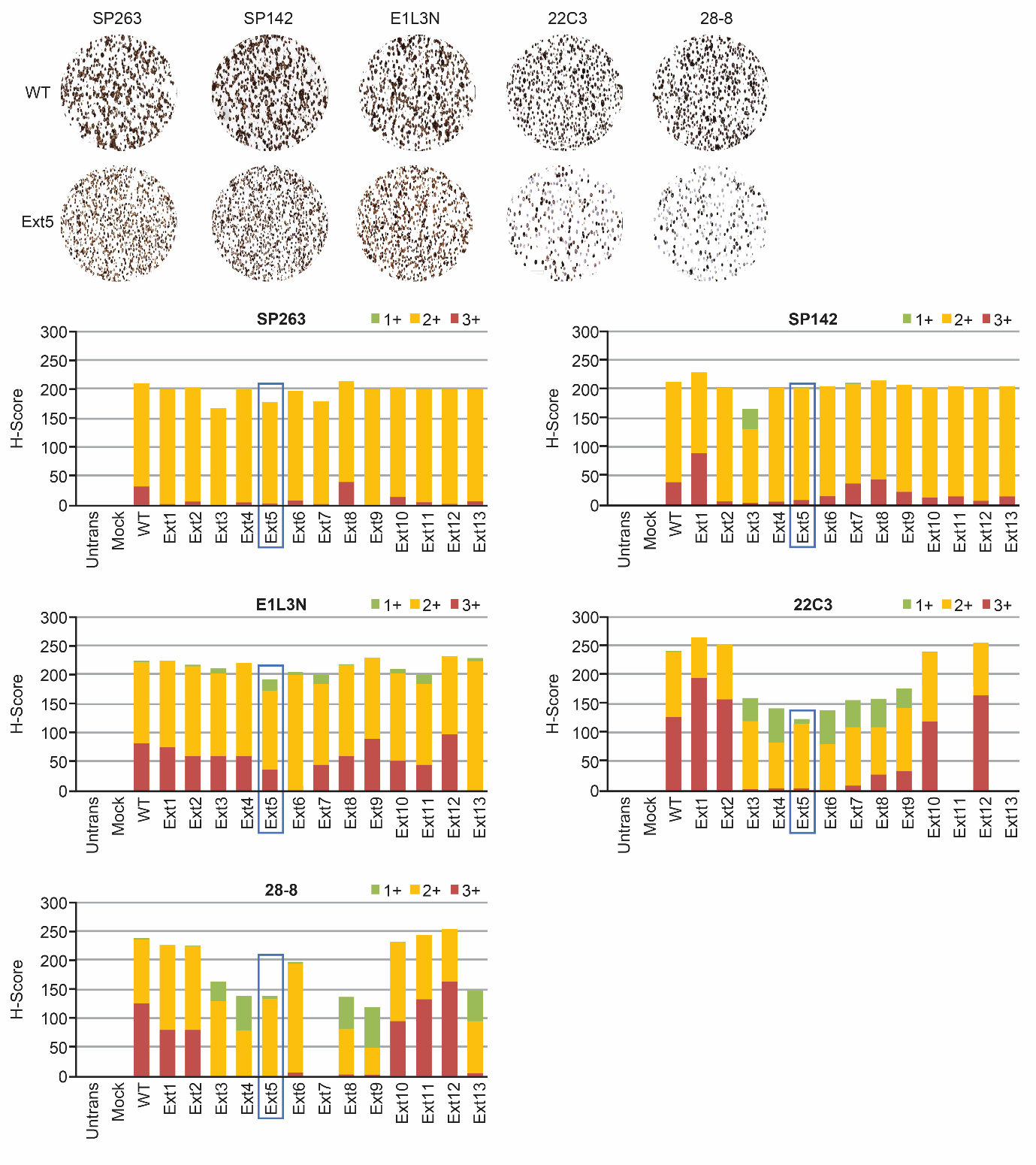


## **Supplementary figure S6.** Deglycosylation of PD-L1. Removal of N-glycosylation by PNGase F results in decreased detection of recombinant PD-L1 by 28-8 and 22C3 antibodies suggesting glycosylation may play a role in binding for these two antibodies. FLAG tag in FLAG 10-His tagged PD-L1 extracellular domain is detected as a control. -, empty lane; D/G, PNGase F-treated PD-L1; G, glycosylated PD-L1; PD-L1, programmed cell death ligand-1.


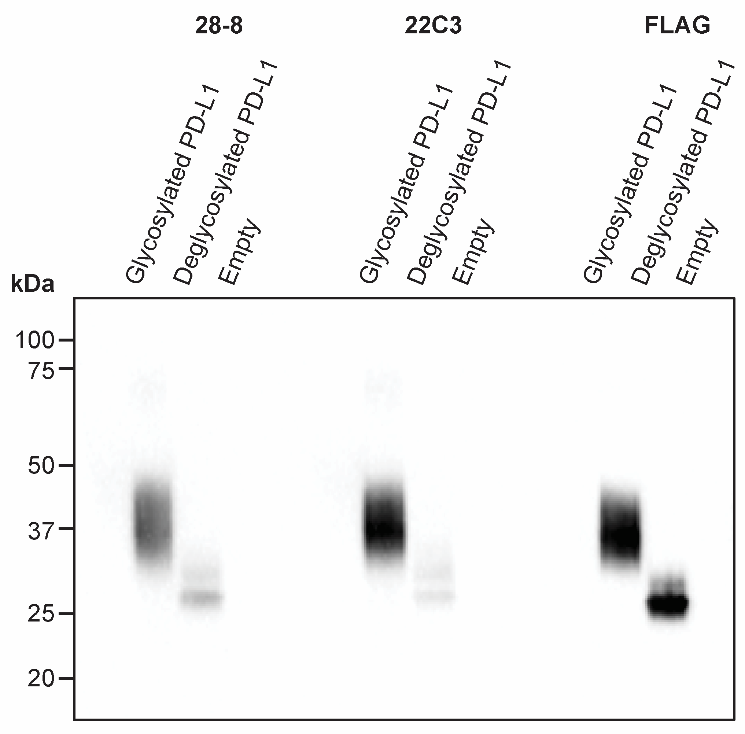


## **Supplementary figure S7**. SP142 demonstrated concordant staining compared with the other PD-L1 antibodies, using an alternative staining protocol. Horizon Discovery CD274 reference standard cell lines (HDX24013) were stained with (A) Dako PD-L1 IHC 22C3 PharmDx assay, (B) Dako PD-L1 IHC 28-8 PharmDx assay, (C) VENTANA PD-L1 (SP263) Assay, (D) VENTANA PD-L1 (SP142) Assay, and (E) SP142 LDT assay protocol on Leica Bond™ automated IHC stainer. Reference standard cell cores 2 and 3 highlight lower PD-L1 staining observed with VENTANA PD-L1 (SP142) Assay compared to VENTANA PD-L1 (SP263) Assay and Dako assays. Staining with an SP142 LDT assay protocol on the Leica Bond™ automated IHC stainer demonstrated that comparable staining to VENTANA PD-L1 (SP263) Assay was achieved (detailed protocol in **Supplementary Appendix F**). This supports the study findings that SP263 & SP142 antibodies are indistinguishable in their recognition of a single epitope sequence and that the observed staining discordance is a result of variance in the assay protocols and not due to antibody epitope. IHC, immunohistochemical; LDT, laboratory developed test; PD-L1, programmed cell death ligand-1.


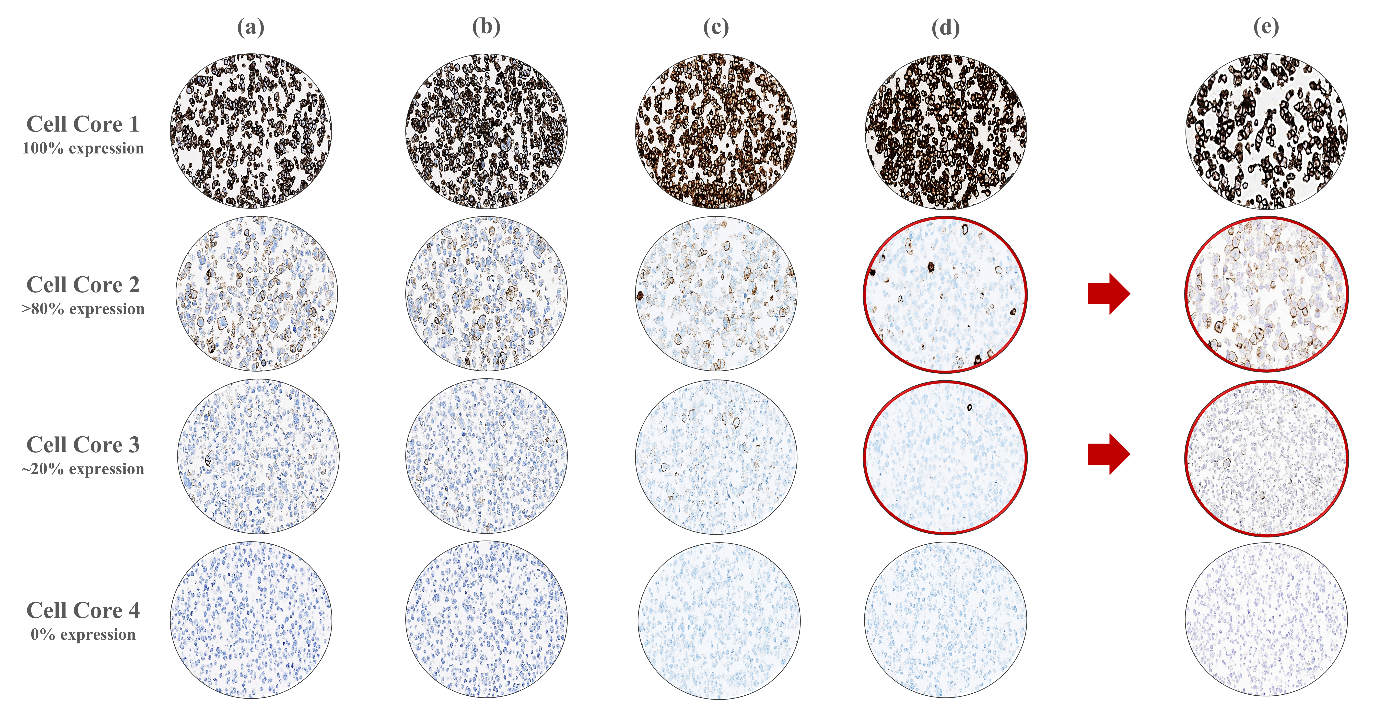

Supplement: Supplementary file 1 — Supplementary material [file 41379_2019_372_MOESM1_ESM.docx]
